# Medication Adherence in the Real World: Lessons from the Diuretic Comparison Project

**DOI:** 10.3390/jcm14165695

**Published:** 2025-08-12

**Authors:** Colleen A. Hynes, Cynthia Hau, Patricia Woods, Sarah Leatherman, Sonia T. Anand, Peter Glassman, Addison Taylor, William C. Cushman, Areef Ishani, Ryan Ferguson

**Affiliations:** 1 Cooperative Studies Program Coordinating Center, VA Boston Healthcare System, Boston, MA 02111, USA; 2 Department of Biostatistics, School of Public Health, Boston University, Boston, MA 02118, USA; 3 Pharmacy Benefits Management Services, Department of Veterans Affairs, Washington, DC 20422, USA; 4 Department of Medicine, VA Greater Los Angeles Healthcare System, Los Angeles, CA 90073, USA; 5 Department of Medicine, David Geffen School of Medicine at UCLA, Los Angeles, CA 90095, USA; 6 Michael E. DeBakey VA Medical Center, Houston, TX 77030, USA; 7 Department of Medicine, Baylor College of Medicine, Houston, TX 77030, USA; 8 Department of Preventive Medicine, University of Tennessee Health Science Center, Memphis, TN 38163, USA; 9 Minneapolis VA Healthcare System, Minneapolis, MN 55417, USA; 10 Department of Medicine, University of Minnesota, Minneapolis, MN 55455, USA; 11 Department of Medicine, Boston University, Boston, MA 02118, USA

**Keywords:** medication adherence, patient characteristics, hypertension, antihypertensive pragmatic trial, older adults, Veterans Affairs

## Abstract

**Background/Objectives**: Antihypertensive treatment is crucial for preventing major adverse cardiovascular events, but suboptimal adherence remains a challenge. **Methods**: This is a secondary analysis of routine care data from a large pragmatic trial comparing two thiazide diuretics: chlorthalidone (CTD) and hydrochlorothiazide (HCTZ). In the trial, 13,523 older hypertensive patients were randomized from 72 Veterans Affairs medical centers. Medication possession ratio (MPR), reflecting adherence to either study medication (CTD or HCTZ), was used and compared across all randomized patients. **Results**: The overall median MPR was 95% for all randomized patients and 80% for 6656 individuals who reached 2.4 years for the average follow-up. Lower MPR was observed in Black, separated, urban-living, and comorbid patients. About 30% of the participants (n = 4022) were categorized as non-adherent using a definition of MPR < 80%. Those with baseline systolic blood pressure ≥ 136, recent smoking history, and prior heart failure and Black participants had decreased odds of having an MPR ≥ 80%, while increased odds of reaching that threshold were observed in those who had an eGFR ≥ 60, received ≥3 antihypertensive medications, were married, or resided in rural areas. **Conclusions**: This analysis provided assessment of real-world medication adherence in a sizable older hypertensive cohort. The proportion of non-adherence found in our analysis was comparable to national trends for US older adults taking blood pressure medications. Identifying sociodemographic characteristics and health conditions associated with non-adherence can help clinicians design targeted interventions for improved adherence to clinically prescribed medications. This is important as hypertension and the older adult population are both expected to grow significantly in the future.

## 1. Introduction

Adherence to antihypertensive therapy (AHT) is essential for controlling hypertension and preventing major adverse cardiovascular events (MACEs), thereby reducing long-term complications and healthcare costs [1,2]. Hypertension affects approximately 70% of older adults in the United States and remains a major modifiable risk factor for cardiovascular (CV) disease [3,4]. AHT is a fundamental component of primary care interventions for hypertension management and MACE prevention [2,5]. Despite its established benefits, non-adherence to prescribed AHT occurs in an estimated 25–50% of patients globally [6]. Given that the older adult population in the United States is projected to increase from 56 million in 2020 to 86 million by 2050, with a corresponding rise in hypertension prevalence, improving adherence to AHT is imperative for effective CV disease prevention [7].

While the relationship between AHT adherence and sociodemographic factors is relatively well documented [8,9], most insights stem from randomized clinical trials (RCTs) or retrospective analyses of electronic health record (EHR) data [10,11]. However, these designs are limited in their ability to capture real-world medication-taking behavior. Patients enrolled in RCTs typically receive enhanced monitoring and support that is not reflective of usual care, while retrospective studies lack prospective data collection and trial randomization. In contrast, pragmatic clinical trials (PCTs) are uniquely positioned to assess medication adherence and its association with medical outcomes in routine clinical settings.

To our knowledge, no prior study has evaluated antihypertensive adherence using prospectively collected EHR data embedded within a randomized, pragmatic trial. Utilizing such data allows for a more accurate reflection of real-world medication use patterns, where pharmacy refill records mirror clinical practice and treatment occurs under usual care conditions [12,13]. PCTs, by maintaining routine healthcare practices while employing gold-standard randomization, offer an opportunity to answer important clinical questions in a real-world context.

The Diuretic Comparison Project (DCP) was a large, nationwide PCT conducted within the Veterans Affairs (VA) Healthcare System that compared two first-line AHTs: hydrochlorothiazide (HCTZ) and chlorthalidone (CTD) [14,15,16]. Over the study period, with the clinically embedded trial approach, the DCP randomized 13,523 hypertensive adults aged ≥65 years and collected real-world clinical data from 72 VA medical centers across the United States. Study medications were prescribed and managed by patients’ primary care clinicians (PCCs) and dispensed via the VA outpatient pharmacy system. EHR-based refill data were captured in near real time and stored in a centralized national database, facilitating the creation of a large, pragmatic dataset for comparative effectiveness research on MACE prevention.

This secondary analysis of the DCP dataset evaluated the association between patient sociodemographic and clinical characteristics and adherence to AHT in routine clinical care. Given the pragmatic design of the DCP, where both arms of randomization reflected usual care and routine pharmacy fills, we combined data on both studied medications to assess overall adherence to AHTs prescribed by their PCCs. We also examined major adverse health outcomes associated with adherence levels to underscore the real-world impact of medication-taking behavior.

## 2. Materials and Methods

### 2.1. Study Design

This secondary analysis leveraged routine care data collected during the multi-center DCP study conducted between 2016 and 2022. The full trial protocol and primary outcomes have been reported previously [14,15]. In brief, the DCP was an open-label PCT designed to assess whether the less commonly prescribed CTD is superior to the more frequently used HCTZ. Both diuretics are considered first-line treatment for hypertension, and their pharmacokinetic characteristics are well-established in the medical literature. The VA Healthcare System contains a flexible EHR infrastructure, allowing researchers to include study-specific computerized physician orders into routine clinical workflows. These features cultivated an opportunity to implement a highly pragmatic clinical trial process, with all study procedures completed through routine patient care or via the existing EHR systems.

Outcome and safety monitoring for the DCP were seamlessly integrated into VA clinical practice. Participants were not required to attend any study-specific clinic visits beyond usual care. Consent, randomization, and trial data collection were accomplished through the successful implementation of electronic study workflows into the existing VA Healthcare System [17,18].

As mentioned, a total of 72 VA medical centers participated in the DCP, enrolling patients from over 500 regional primary care clinics affiliated with the main medical centers. Administrative, patient, and clinical data were recorded at each individual clinic through localized EHR systems. Such data were transferred daily from each VA healthcare site to a centralized data repository, the VA Corporate Data Warehouse (CDW) [19].

The DCP was approved by the VA Central Institutional Review Board (CIRB) (ClinicalTrials.gov, trial number NCT02185417). EHR-based assent was collected from PCCs prior to randomization, and verbal consent was obtained for all eligible patients who agreed to participate.

This secondary analysis was deemed exempt from human subject requirement research, as it utilized de-identified data and involved no interaction with DCP participants. Accordingly, review and approval by the VA CIRB were not required.

### 2.2. Adherence Data

To preserve the pragmatic nature of the trial, in which the study intervention was embedded in usual clinical environments, PCCs were informed that they retained their right as the decision-makers for antihypertensive treatment decisions [17]. PCCs were permitted to modify randomized treatment regimens as clinically indicated, including dose adjustments, medication substitutions, or discontinuations. Prescriptions for HCTZ and CTD were ordered by the PCCs through the local EHR system, and all prescribing data were captured in the VA CDW.

Patients who remained on HCTZ continued to receive their medication through their existing prescription [15]. For patients randomized to CTD, HCTZ was discontinued and an equipotent dose of CTD was dispensed by the local VA pharmacy [14]. Upon approval, these archived data in the CDW were accessible to the researchers for study purposes [20].

### 2.3. Medication Adherence Assessment

The medication possession ratio (MPR) is a formula commonly used to measure adherence by evaluating medication refill patterns using pharmacy data documented at the clinic level [21,22]. Medication adherence was quantified using the MPR, based on pharmacy dispensing records for CTD and HCTZ fills during follow-up. The MPR was calculated as the total number of days the study medication was supplied divided by the total follow-up time. A threshold of ≥80% was used to classify patients as adherent [23].

### 2.4. Patient Characteristics

Baseline demographics included age, sex, race, ethnicity, and marital status and were based on self-reported information. Patients’ residential setting (urban/rural) and baseline smoking status (never/former/current) were determined using VA standard definitions [24,25]. Clinical characteristics such as baseline estimated glomerular filtration rate (eGFR), systolic blood pressure (SBP), and medical histories (heart failure [HF], myocardial infarction [MI], stroke, and diabetes) were extracted from inpatient, outpatient, and fee basis records.

Medical conditions were identified using the International Classification of Diseases (ICD) diagnosis codes and were defined as any documented occurrence prior to randomization. Concurrent AHT use was identified through outpatient pharmacy records and measured as any prescription fill within 182 days before randomization.

### 2.5. Other Follow-Up Measures

Follow-up measures of interest included outpatient SBP readings, inpatient hospitalizations, all-cause deaths, and the main study outcomes, encompassing non-fatal major CV events (hospitalization for HF, MI, stroke, and urgent coronary revascularization for unstable angina). These data were obtained from national databases, including the VA CDW and the Medicare and National Death Index (NDI). In particular, events and deaths that occurred outside the VA were captured via linked data from Medicare inpatient claims and the NDI. Major CV events and deaths were validated with approved electronic algorithms, and manual adjudication was used in instances where outcome diagnosis was indeterminate [18]. SBP measurements were extracted from routine outpatient clinical visits.

The use of other medications was also assessed through VA pharmacy records, and we focused on agents related to the DCP trial outcome and safety measures, including outpatient pharmacy fills for allopurinol, sodium–glucose cotransport protein 2 inhibitors (SGLT2i), and potassium supplementation.

### 2.6. Statistical Analysis

Adherence rates (MPR ≥ 80% vs. <80%) were summarized by patient baseline characteristics using frequencies and percentages. The median MPR values with the interquartile range (IQR) were calculated across subgroups, and differences were compared via Wilcoxon rank-sum tests. Characteristics with significant associations (*p*  < 0.05) were entered into a multivariable logistic regression model (adherence: MPR ≥ 80% as outcome) using a stepwise backward selection method. Variables with univariate *p* < 0.15 were included initially, with removal criteria set at *p* ≥ 0.05. Major CV outcomes, hospitalizations, and mortality were described using annualized event rates and mean (SD) events per patient. Analyses were conducted with SAS version 9.4 (SAS Institute).

## 3. Results

### 3.1. Patient Characteristics and Adherence

Among the 13,523 randomized patients, the overall median MPR was 95%, with significant differences across race, marital status, residency, smoking status, history of stroke, heart failure, baseline eGFR, SBP, and number of concurrent AHT prescriptions (Table 1). A total of 6656 patients reached the median follow-up of 2.4 years, with the median MPR ranging from 83% to 94% across baseline characteristics. The MPRs were statistically different between demographic groups, both in the total cohort and in the subset containing those who reached median follow-up. These included race, marital status, rural residency, smoking status, eGFR condition, and baseline history of stroke and HF.

**Figure 1:**
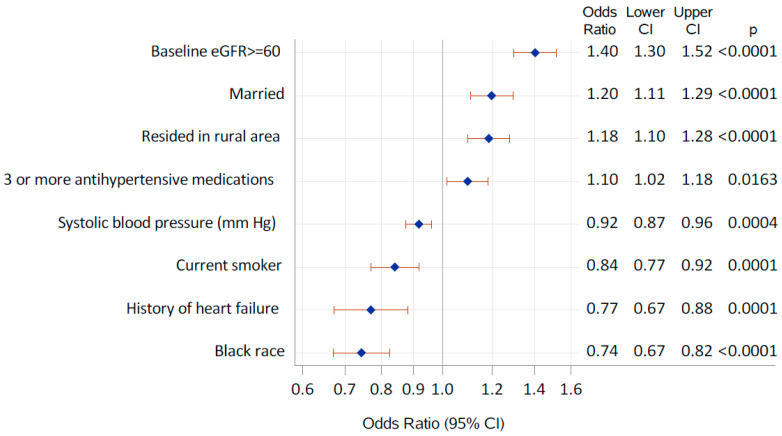
Factors associated with the odds of having a medication possession ratio (MPR) ≥80%.

In the multivariable logistic regression, lower odds of achieving MPR ≥ 80% were observed in patients with higher baseline SBP, those who were current smokers, those with a history of HF, and those who were Black. Conversely, higher odds were noted in those with eGFR ≥ 60, those with a marital status of married, those with rural residency, and those who had used ≥3 antihypertensive agents (Figure 1).

### 3.2. Follow-Up Measures of Interest

A total of 9501 (70.3%) of patients met the adherence threshold of MPR ≥ 80%, and 4022 (29.7%) were considered non-adherent (Table 2). The number of patients with recorded SBP measurements was similar between the adherent and non-adherent groups; however, the mean number of SBP readings was higher among non-adherent participants. The mean SBP was consistently lower in adherent patients.

The frequency of all-cause hospitalization and non-fatal major CV and mortality events was markedly higher among non-adherent patients. The number of hospitalized patients was nearly twice as high in the non-adherent group, and major CV events and deaths occurred more frequently in this group. The use of other relevant medications was similar between the adherence groups (Supplemental Table S1).

## 4. Discussion

Pragmatic clinical trials, such as the DCP, offer a valuable opportunity to assess medication adherence under real-world conditions. Given the high burden of hypertension among older US adults, identifying adherence patterns is critical for optimizing health outcomes and quality of life [26]. To our knowledge, this study represents the first assessment of medication adherence patterns within a PCT using embedded EHR data.

Our observed overall average adherence rate and adherence achievement by 70% of participants were comparable to rates observed in traditional RCTs [27,28]. These findings reinforce the idea that pragmatic adherence assessments can yield reliable, clinically relevant data for effectiveness research. Importantly, identifying characteristics associated with poor adherence allows clinicians to target interventions toward high-risk subgroups.

Overall, we found non-adherence in the DCP (29.7%) to be comparable to national trends of non-adherence among insured US older adults taking blood pressure medication (24% to 28%) and in adults taking diuretics specifically (33%) [29]. Also consistent with national trends, non-adherence was more common among patients aged ≥75 years, potentially reflecting greater regimen complexity and age-related impairments [29,30]. Racial disparities in adherence rates were also observed, aligning with the literature linking minority status to poorer health outcomes and socioeconomic disadvantages [31,32,33,34]. Additionally, non-adherence was more prevalent among those with a history of CV events and higher baseline SBP, factors known to complicate hypertension management [35,36,37,38,39].

We confirmed that non-adherence was associated with an increased risk of hospitalization, CV events, and mortality—findings that are consistent with prior studies [2,40,41]. Given that the clinical effectiveness of AHT depends on adherence, these results underscore the importance of proactive adherence monitoring and support in typical care settings.

All participants in the DCP cohort were receiving HCTZ at baseline for the treatment of hypertension (HTN), indicating an established antihypertensive regimen. Consequently, adherence in this population may have been higher than in individuals with newly diagnosed HTN initiating therapy, among whom first-year non-persistence rates have been reported to range from 30% to 80% [8]. Additionally, adherence among the DCP participants may have been influenced by their awareness of their participation in a clinical trial. These analyses do have some limitations. One limitation of our assessment is that the population of the DCP predominantly consisted of older White males, limiting generalizability to other populations. Reliance on pharmacy refill data using the MPR as the only metric leads to the assumption of medication ingestion, which may not always reflect actual patient behavior. However, its observed association with clinical outcomes in our study supports its validity as a proxy measure. While other studies have combined the MPR with pill counts or patient-reported adherence through questionnaires or surveys, the pragmatic design and large sample size precluded collection of self-reported adherence data. Additionally, undocumented medication discontinuations and inpatient hospitalizations could lead to the misclassification of adherence.

The strengths of this study include the large, nationally representative VA cohort, the use of longitudinal EHR-based pharmacy data, and the pragmatic trial design, reflecting routine care practices. Our identification of adherence-associated factors provides actionable insights for primary care clinicians to strengthen adherence monitoring and improve patient support. Moreover, as adherence was measured by assessing fill rates, one potential means of addressing and perhaps improving adherence in clinic settings would be for clinicians to review the timing between fills, which can readily be achieved with the VA’s EHR, and to discuss the clinical implications of late fills with patients. Primary care teams could also routinely screen for potential adherence barriers.

Adherence could be improved through the use of other effective tools, such as pharmacy-based interventions and digital tools like smartphone applications (apps), telemedicine, and electronic medication packages [42]. These technologies enable real-time adherence monitoring and can provide behavioral prompts to patients.

In the era of artificial intelligence and its integration into healthcare, we anticipate the emergence of novel technologies that will enhance adherence through patient prediction analysis and the personalization of HTN management plans [43]. While these technologies are not a substitute for clinician–patient interaction, they represent a growing set of tools that can complement individualized treatment strategies and enhance adherence.

## 5. Conclusions

This pragmatic analysis of the DCP demonstrated that real-world adherence rates to antihypertensive medications are consistent with those reported in traditional RCTs. The patient characteristics, including race, age, marital status, and comorbidities, significantly influenced adherence. Our findings emphasize the need for targeted, proactive adherence interventions in routine care. The alignment of our results with the existing literature highlights the robustness of pragmatic trials for informing real-world clinical practice and improving medication utilization outcomes. Although adherence among the DCP was relatively favorable, adherence remains a persistent challenge in hypertension management. Traditional monitoring approaches could be complemented by incorporating digital technologies, offering a promising path forward for improving adherence and patient outcomes.

## Figures and Tables

**Table 1:** Effects of patient characteristics and baseline clinical factors on medication possession ratio (MPR).

Parameter	Category	Total Randomized		Adherence		6656 Randomized with ≥2.4 yrs of Follow-Up		Adherence	
		N	% of N	MPR% (IQR)	*p*	n	% of n	MPR% (IQR)	*p*
Age	<72	6751	49.9	94.8 (72.6–101.8)	0.108	3774	56.7	92.7 (66.3–100.1)	0.449
	≥72	6772	50.1	95.1 (72.5–102.1)		2882	43.3	92.4 (62.7–100.2)	
Sex	Female	431	3.2	95.7 (71.0–103.0)	0.109	168	2.5	93.5 (67.6–64.5)	0.396
	Male	13,092	96.8	95.0 (72.6–101.9)		6488	97.5	92.6 (64.5–100.1)	
Race	Other ^1^	324	2.4	92.7 (69.0–100.6)		180	2.7	90.3 (68.0–99.0)	
	Black ^2^	2027	15.0	89.8 (64.2–100.6)	<0.001 *	1039	15.6	87.8 (57.8–99.0)	<0.001 *
	White	10,454	77.3	95.7 (75.0–102.1)		5107	76.7	93.4 (66.5–100.4)	
	Unknown	718	5.3	94.4 (72.3–101.7)		330	5.0	92.2 (64.8–99.6)	
Ethnicity	Not Hispanic/Latino	12,549	92.8	95.1 (72.8–101.9)	0.072	6191	93.0	92.7 (64.5–100.2)	0.496
	Hispanic/Latino	494	3.7	91.6 (67.6–101.6)		231	3.5	90.8 (66.1–99.9)	
	Unknown	480	3.6	95.3 (70.6–102.0)		234	3.5	92.0 (63.9–100.0)	
Marital status	Married	8560	63.3	95.7 (75.6–102.2)	<0.001 *	4170	62.7	93.6 (68.1–100.4)	<0.001 *
	Separated ^3^	4026	29.8	93.1 (67.4–101.3)		2017	30.3	90.1 (59.8–99.5)	
	Single	850	6.3	94.4 (69.9–101.7)		430	6.5	93.1 (62.7–100.6)	
	Unknown	87	0.6	91.7 (65.6–103.6)		39	0.6	91.9 (52.9–102.5)	
Residency	Urban	7375	54.5	93.8 (69.3–101.8)	<0.001 *	3720	55.9	91.1 (61.5–100.0)	<0.001 *
	Rural	6122	45.3	95.9 (76.7–102.1)		2923	43.9	94.3 (69.7–100.4)	
	Unknown	26	0.2	89.6 (69.2–97.4)		13	0.2	89.6 (49.5–96.7)	
Baseline smoking status	Never	3486	25.8	94.7 (73.8–101.8)	<0.001 *	1716	25.8	92.5 (66.1–100.0)	<0.001 *
	Former	5840	43.2	95.7 (74.6–102.0)		3052	45.9	93.6 (67.3–100.4)	
	Current	2957	21.9	93.2 (67.8–101.4)		1432	21.5	89.8 (60.8–99.8)	
	Unknown	1240	9.2	95.3 (74.2–102.8)		456	6.9	92.5 (57.6–100.0)	
History of MI	No	13,035	96.4	95.0 (72.8–101.9)	0.692	6444	96.8	92.7 (65.1–100.2)	0.017 *
	Yes	488	3.6	93.6 (65.5–103.3)		212	3.2	86.3 (49.0–99.1)	
History of stroke	No	12,494	92.4	95.1 (73.1–101.9)	0.034 *	6147	92.4	92.7 (65.6–100.2)	0.039 *
	Yes	1029	7.6	93.4 (64.6–101.9)		509	7.7	90.3 (55.8–99.9)	
History of heart failure	No	12,472	92.3	95.1 (73.4–101.9)	0.003 *	6184	92.9	92.8 (65.6–100.2)	0.001 *
	Yes	1051	7.8	92.7 (62.6–102.1)		472	7.1	87.3 (52.7–99.3)	
History of diabetes	No	7494	55.4	94.9 (72.9–101.6)	0.249	3733	56.1	92.7 (64.6–99.9)	0.441
	Yes	6029	44.6	95.1 (72.2–102.2)		2923	43.9	92.4 (64.2–100.4)	
Baseline eGFR	Stage 1–2	9038	66.8	95.7 (76.5–101.9)	<0.001 *	4742	71.2	93.8 (69.5–100.3)	<0.001 *
	Stage 3a	2234	16.5	93.6 (68.3–102.1)		1012	15.2	89.1 (59.9–100.1)	
	Stage 3b	710	5.3	91.7 (58.1–101.0)		320	4.8	86.7 (48.1–98.2)	
	Stage 4–5	283	2.1	91.2 (54.6–101.6)		141	2.1	83.2 (46.7–98.9)	
	Unknown	1258	9.3	93.0 (63.7–101.9)		441	6.6	90.1 (55.7–99.7)	
Baseline SBP	<136	6449	47.7	95.4 (75.1–102.1)	0.006 *	3139	47.2	93.2 (67.6–100.2)	0.056
	≥136	7074	52.3	94.5 (70.7–101.9)		3517	52.8	91.9 (62.5–100.1)	
Antihypertensive prescription	<3	6391	47.3	94.3 (71.3–101.3)	<0.001 *	3205	48.2	92.3 (64.3–99.7)	0.069
	≥3	7132	52.7	95.6 (74.0–102.4)		3451	51.9	92.9 (64.7–100.5)	

^1^ Other is based on Veterans self-reported racial identification. ^2^ Black denotes Veterans who identified as Black or African American. ^3^ Status of separated includes those who were separated, divorced, or widowed. * *p* ≤ 0.05.

**Table 2:** Follow-up measures of interest.

	Year 1		Year 2		Year 3		Year 4		Year 5	
	Adherent N = 9501	Non-Adherent N = 4022	Adherent N = 8288	Non-Adherent N = 3632	Adherent N = 5128	Non-Adherent N = 2586	Adherent N = 2898	Non-Adherent N = 1633	Adherent N = 747	Non-Adherent N = 456
No. (%) patients with SBP measure	8674 (91.3)	3710 (92.2)	6583 (79.4)	2902 (79.9)	4311 (84.1)	2111 (81.6)	2047 (70.6)	1151 (70.5)	542 (72.6)	318 (69.7)
Mean (SD) no. of SBP records	5.2 (5.0)	6.6 (6.7)	4.5 (4.6)	6.0 (7.0)	4.7 (5.2)	6.5 (9.2)	4.3 (5.0)	5.7 (7.5)	3.8 (3.8)	6.1 (16.8)
Mean (SD) SBP measure	138.4 (13.2)	140.3 (14.7)	138.8 (13.8)	140.9 (15.8)	139.0 (14.4)	141.8 (16.0)	139.3 (15.0)	142.4 (17.2)	139.1 (15.1)	140.2 (15.9)
No. (%) patients who had hospitalizations	946 (10.0)	903 (22.5)	738 (8.9)	743 (20.5)	478 (9.3)	482 (18.6)	188 (6.5)	205 (12.6)	47 (6.3)	53 (11.6)
Mean (SD) no. of hospitalizations	1.4 (0.9)	2.0 (1.5)	1.5 (1.0)	2.0 (1.6)	1.4 (1.0)	1.9 (1.6)	1.4 (0.7)	1.7 (1.3)	1.5 (0.8)	1.9 (1.9)
No. (%) patients had major CV events	117 (1.2)	236 (5.9)	96 (1.2)	238 (6.6)	75 (1.5)	132 (5.1)	31 (1.1)	48 (2.9)	5 (0.7)	15 (3.3)
Mean (SD) no. of major CV events	1.3 (0.8)	1.5 (1.0)	1.4 (0.9)	1.6 (1.0)	1.3 (0.9)	1.5 (1.2)	1.1 (0.3)	1.5 (1.1)	1.8 (0.8)	1.2 (0.4)
No. (%) deceased patients	181 (1.9)	93 (2.3)	167 (2.0)	105 (2.9)	114 (2.2)	108 (4.2)	49 (1.7)	52 (3.2)	10 (1.3)	13 (2.9)

## Data Availability

The datasets generated and analyzed during the current analysis and DCP are not publicly available but are available on request with an IRB-approved protocol and upon completion of a VA-approved data use agreement.

## Supplementary Materials

The following supporting information can be downloaded at https://www.mdpi.com/article/10.3390/jcm14165695/s1: Supplemental Table S1: The use of selected medications over time.

## Abbreviations

The following abbreviations are used in this manuscript:

AHT	Antihypertensive therapy
BP	Blood pressure
CTD	Chlorthalidone
CV	Cardiovascular
DCP	Diuretic comparison project
eGFR	Estimated glomerular filtration rate
EHR	Electronic health record
HCTZ	Hydrochlorothiazide
HF	Heart failure
MACE	Major adverse cardiovascular event
MI	Myocardial infarction
MPR	Medication possession ratio
PCC	Primary care clinician
PCT	Pragmatic clinical trial
RCT	Randomized control trial
SBP	Systolic blood pressure
SGLT2i	Sodium–glucose cotransport protein 2 inhibitors
VA	Veterans Affairs
