# Supplementary material for: Medication Adherence in the Real World: Lessons from the Diuretic Comparison Project
Source: J Clin Med. 2025 Aug 12;14(16):5695. doi: 10.3390/jcm14165695 (PMC12386936; doi:10.3390/jcm14165695)
Supplement: Supplementary file 1 [file jcm-14-05695-s001.zip › jcm-3737312-supplementary.pdf]

Supplemental Table S1. Use of selected medications over time

Adherent (N=9,501)	Baseline		6 months		12 months		18 months		24 months	
	Adherent (n=9,501)	Non-adherent (n=4,022)	Adherent (n=9,501)	Non-adherent (n=4,022)	Adherent (n=9,409)	Non-adherent (n=3,989)	Adherent (n=8,288)	Non-adherent (n=3,632)	Adherent (n=6,676)	Non-adherent (n=3,103)
**Allopurinol**	754 (7.9)	313 (7.8)	782 (8.2)	318 (7.9)	783 (8.3)	330 (8.3)	686 (8.3)	290 (8.0)	570 (8.5)	241 (7.8)
**SGLT2i**	264 (2.8)	85 (2.1)	380 (4.0)	140 (3.5)	480 (5.1)	182 (4.6)	411 (5.0)	174 (4.8)	357 (5.3)	182 (5.9)
**Potassium Supplementation**	1,302 (13.7)	485 (12.1)	1,412 (14.9)	536 (13.3)	1,471 (15.6)	579 (14.5)	1,262 (15.2)	514 (14.2)	1,037 (15.5)	474 (15.3)
Potassium chloride	1,066 (11.2)	411 (10.2)	1,154 (12.1)	459 (11.4)	1,204 (12.8)	469 (11.8)	1,038 (12.5)	384 (10.6)	856 (12.8)	341 (11.0)
Potassium citrate	75 (0.8)	11 (0.3)	77 (0.8)	10 (0.2)	71 (0.8)	14 (0.4)	55 (0.7)	8 (0.2)	37 (0.6)	10 (0.3)
Potassium sparing	192 (2.0)	70 (1.7)	213 (2.2)	89 (2.2)	232 (2.5)	134 (3.4)	204 (2.5)	156 (4.3)	172 (2.6)	153 (4.9)

SGLT2i = sodium-glucose cotransport protein 2 inhibitors
